# Knowledge, attitudes and practices regarding the Pap test among women in northeastern Brazil: Conhecimentos, atitudes e práticas sobre o exame de Papanicolaou em mulheres do nordeste brasileiro

**DOI:** 10.1590/1516-3180.2014.1321551

**Published:** 2014-02-01

**Authors:** Carla Lorenna Ferreira de Albuquerque, Marla da Paschoa Costa, Felipe Moreira Nunes, Roberto Wagner Junior Freire de Freitas, Paulo Roberto Medeiros de Azevedo, José Veríssimo Fernandes, Juciane Vaz Rego, Humberto Medeiros Barreto

**Affiliations:** I Nursing Undergraduate, Department of Health Sciences, Universidade Federal do Piauí (UFPI), Floriano, Piauí, Brazil; II MSc. Professor, Department of Health Sciences, Universidade Federal do Piauí (UFPI), Floriano, Piauí, Brazil; III PhD. Professor, Department of Statistics, Universidade Federal do Rio Grande do Norte (UFRN), Natal, Rio Grande do Norte, Brazil; IV PhD. Professor, Department of Parasitology and Microbiology, Universidade Federal do Rio Grande do Norte (UFRN), Natal, Rio Grande do Norte, Brazil

**Keywords:** Papillomavirus infections, Uterine cervical neoplasms, Vaginal smears, Women's health, Socioeconomic factors, Infecções por papillomavirus, Neoplasias do colo do útero, Esfregaço vaginal, Saúde da mulher, Fatores socioeconômicos

## Abstract

**CONTEXT AND OBJECTIVE:**

The Papanicolaou (Pap) test has been shown to be effective in preventing cervical cancer. However, both the national and international literature shows that Pap testing has not reached the level of coverage desired. The objective of this study was to assess women's knowledge, attitudes and practices regarding the Pap test and to investigate whether there are any associations between these three factors and the women's sociodemographic characteristics.

**DESIGN AND SETTING:**

Cross-sectional descriptive study conducted in Floriano, Piauí.

**METHODS:**

: The study was conducted among 493 women between November 2009 and December 2010. A questionnaire with precoded questions was used, and the responses were analyzed in terms of appropriateness in relation to the Pap test.

**RESULTS:**

: The degrees of adequacy of knowledge, attitudes and practices regarding the Pap test were 36.7%, 67.2% and 69.6%, respectively. Among the main barriers against testing, absence of symptoms and a sense of embarrassment were the most notable.

**CONCLUSIONS:**

Women who visit doctors periodically had the most appropriate practices regarding the Pap test, but their knowledge of the procedure was poor. This suggests that these women were not receiving adequate information about the benefits of periodic testing.

## Abstract (pt)

**CONTEXTO E OBJETIVO:**

O exame de Papanicolaou já mostrou efetividade na prevenção de câncer do colo do útero. A literatura nacional e internacional tem mostrado que o exame de Papanicolaou não tem alcançado o índice de cobertura desejado. O objetivo deste estudo foi avaliar os conhecimentos, atitudes e práticas entre mulheres em relação ao exame de Papanicolaou e verificar se existe associação entre esses comportamentos e as características sociodemográficas.

**TIPO DE ESTUDO E LOCAL:**

Estudo descritivo e transversal realizado em Floriano, Piauí.

**MÉTODOS:**

O estudo foi conduzido com 493 mulheres no período de novembro de 2009 a dezembro de 2010. Utilizou-se um questionário com perguntas precodificadas, cujas respostas foram analisadas quanto à adequação dos comportamentos em relação ao exame.

**RESULTADOS:**

Os graus de adequação dos conhecimentos, atitudes e práticas em relação ao exame foram de 36,7%, 67,2% e 69,6%, respectivamente. Dentre as principais barreiras para a sua realização, destacaram-se a ausência de sintomas e a vergonha.

**CONCLUSÃO:**

As mulheres que realizam consultas periodicamente apresentam prática mais adequada, porém com baixa adequação de conhecimentos frente ao procedimento, sugerindo que não estejam recebendo as informações adequadas sobre os benefícios da realização periódica do exame de Papanicolaou.

## INTRODUCTION

Cervical cancer is the second biggest cause of cancer-related deaths among women worldwide, but the incidence is higher in developing countries.^1^ ^,^ ^2^ In Brazil, this disease remains the third most common malignant neoplasm after non-melanoma skin cancer and breast cancer.^3^ ^,^ ^4^ In 2012, the approximate incidence rate of cervical cancer per 100,000 women was 17.49 for Brazil, 17.96 for northeastern Brazil and 22.58 for the state of Piauн.^3^

The etiology of cervical cancer is directly related to persistent infection of the uterine cervix with human papillomavirus (HPV) genotypes that have a high oncogenic potential.^5^ ^,^ ^6^ HPV infection is considered to be a necessary but insufficient cause of development of neoplasms or their precursor lesions, because viral deoxyribonucleic acid is present in 99.7% of cervical cancer cases.^7^ ^-^ ^9^

Importantly, this malignant neoplasm is one of many cancers with great potential for prevention and cure. The progression of cervical cancer is relatively slow, passing through various stages of precancerous intraepithelial lesions before advancing to its invasive form.^10^ This characteristic of the disease, combined with the relative ease of diagnosis, has allowed physicians to detect this cancer during its earliest stages, when treatment results in a high cure rate.^10^ ^,^ ^11^ Moreover, the infectious nature of cervical cancer's etiological agent has made implementation of preventive measures possible, including active immunization against HPV genotypes with higher oncogenic potential.^7^ ^,^ ^11^ ^,^ ^12^

The Papanicolaou (Pap) test is a simple method for detecting morphological changes in the uterine cervix from desquamated epithelial cells. Because the test is quick, painless, broadly applicable, easy to perform, performable in outpatient clinics and inexpensive, it has been considered to be the best method for cervical cancer screening.^10^ ^,^ ^13^ ^,^ ^14^ However, an estimated 40% of Brazilian women have never been tested.^15^ Low compliance arises for many reasons, including difficulties in accessing healthcare services, emotional discomfort for some women, embarrassment, social taboos, socioeconomic conditions and poor understanding of the benefits of testing for preventing cervical cancer.^16^ These barriers have hindered achievement of the desired level of test coverage. Information concerning test coverage and the factors associated with test noncompliance among women in northeastern Brazil is still scarce.

## OBJECTIVE

The purpose of the present study was to assess the knowledge, attitudes and practices of women in the city of Floriano, Piauн, regarding the Pap test, and to determine whether there was any association between the appropriateness of these three factors and the sociodemographic characteristics and other variables of this population.

## METHODS

A cross-sectional descriptive study was conducted in the city of Floriano, Piauн, from November 2009 to December 2010, through home visits and interviews using a standardized questionnaire. The study included 493 women between 15 and 69 years of age (mean: 35.4 years) residing in both the urban and the rural areas of the municipality. 

The urban center of the municipality is located 240 km from the state capital and the total estimated population of the municipality is 57,690 inhabitants, of whom 49,970 live in urban areas, 7,720 live in rural areas and 30,381 are female. The primary economic activities include agriculture, raising livestock, extracting natural resources and trade. Most of the population is poor and depends on the public health system, which consists of one 97-bed hospital, 25 primary healthcare units and 24 teams within the Family Health Program.

A sample size calculation was used to determine the number of women to be interviewed. This calculation used a statistical method based on the demographics of the municipality's female population and taking into consideration both rural and urban areas. The variable considered for defining the sample calculation was *p* equals the proportion of women between 20 and 59 years of age within the population of women aged over 15 years. In this case, the calculation of sample size was given by:

$$n=\frac{Np\left(1-p\right)}{ND+p\left(1-p\right)}$$

where *n* equals the sample size, *N* equals the total number of women aged over 15 years in the city and *D* equals ε^2^/4, such that e is a boundary error estimation of p, which satisfies P(| p- $\hat{p}$| < ε) = 0.95, in which $\hat{p}$is an estimate for p. According to a survey conducted by the Municipal Health Department, N = 23,318 women and a preliminary estimate for p was given by $\hat{p}$= 0.72. By taking ε = 0.04 and inserting these values to determine n in the formula above, it was found that n = 493 women.

Three interviewers were trained to administer the survey and collect data. The research project and its objectives were explained to potential participants and assurances were given that confidential information would be safeguarded. These potential participants were then asked whether they would like to participate in the study. Those who voluntarily agreed to participate then signed an informed consent form and answered the questions on the questionnaire. The questions, which were designed to evaluate the three factors, consisted of direct questions in which the participants were asked to state their age, place of residence, education level, marital status, ethnicity, religion, family income, number of visits to a doctor during the previous year, sexual activity, use of contraceptives and parity. Only the women who voluntarily decided to participate in the study gave responses to the questionnaire and therefore there were no sample losses. The questions were asked by the interviewer orally without inducing responses.

To analyze the data we adopted the following definitions:

- Inadequate knowledge: a situation in which the women claimed that they had heard of the test but did not know the reason why the test was performed.
- Inappropriate attitude: a situation in which the women considered testing unnecessary or had no opinion about receiving the test.
- Inadequate practice: a situation in which the women claimed to have been tested more than three years ago, to have been tested only once in their lifetime or to have never been tested.

The data collected were independently digitized twice and were stored in an Excel database. Statistical analyses were performed using the Statistical Package for the Social Sciences (SPSS) software, version 16.0. To determine whether there was any association between variables, the chi-squared test was used, and a P-value < 0.05 was considered significant.

This research was submitted for approval to the Ethics Committee for Human Research of Universidade Federal do Piauн and was approved under the protocol number 0156.0.045.000-09.

## RESULTS

Analysis of the data collected from the questionnaire allowed a profile to be created for the study participants. All of the women in the study were between 15 and 69 years of age (mean: 35.4 ± 14.3 years), and most were between 20 and 45 years of age. Most of the women lived in the urban areas of the municipality, possessed only elementary school education, possessed a monthly family income at or below the minimum wage level, professed Catholicism as their religion, engaged in an active sex life, were married or living in a stable relationship with their partner and had one to three children.

In terms of the degree of knowledge about the test, 94.5% of the women interviewed had heard of the procedure, but only 36.7% had adequate knowledge of the test. The doctor was cited as the primary source of information about the test by 44.2% of the participants, while friends or relatives were cited by 19.5% of the participants (Table 1).

**Table 1:**
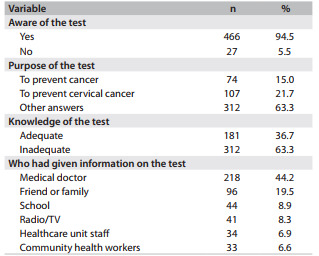
Sources of information and adequacy of knowledge relating to the Pap test in the municipality of Floriano, Piauí, Brazil, 2010 (n = 493)

All of the women interviewed considered the test necessary, but only 67.1% had an appropriate attitude regarding the procedure, through expressing conscious recognition of its advantages and benefits and correctly indicating the reasons for periodically undergoing the test. Among those who had an appropriate attitude, 46.7% justified the need for the test as a means of preventing cervical cancer, and 20.5% justified the test as a means of preventing cancer but without specifying the type of cancer prevented (Table 2).

**Table 2:**
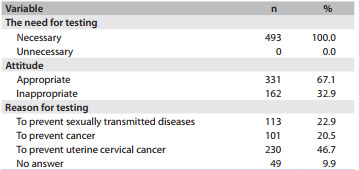
Attitudes towards and reasons for undergoing the Pap test reported by women in the municipality of Floriano, Piauí, Brazil, 2010 (n = 493)

With regard to whether the participants had ever undergone the test, 75.9% of them reported undergoing the procedure at some point, while 24.1% reported never having undergone the test. Among those who said that they had undergone the procedure, 69.6% said that they had been tested at a frequency of at least once every three years, thus demonstrating an appropriate level of test practice, considering that this frequency is within the acceptable limits recommended by the Brazilian Ministry of Health. The primary barriers against adequate frequency of testing were reported to be absence of symptoms (39.5%) and being too embarrassed (26.9%) to undergo the procedure (Table 3).

**Table 3:**
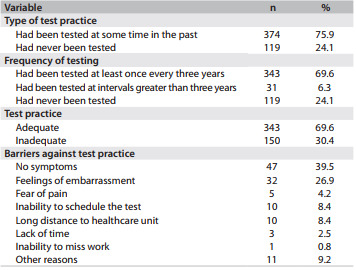
Test practices, adequacy of test practices and barriers against Pap testing reported by women in the municipality of Floriano, Piauí, Brazil, 2010 (n = 493)

Statistically significant associations between knowledge of the test and age, social class, education level and family income level were observed (Table 4). The appropriateness of the women's attitude towards the test was associated with age, schooling level, marital status, sexual activity, frequency of consultations with doctors and parity. The adequacy of test practice presented associations with age, sexual activity, numbers of consultations with doctors, contraception and parity.

**Table 4:**
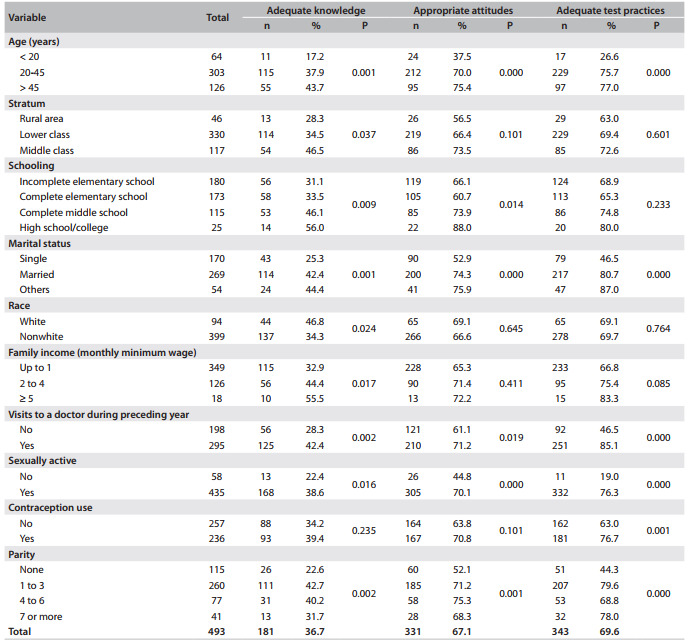
Assessment of adequacy of knowledge, attitudes and practices relating to the Pap test, according to sociodemographic and reproductive characteristics, among women in the municipality of Floriano, Piauí, Brazil, 2010 (n = 493)

## DISCUSSION

Among the 493 women included in our study, most were between 20 and 45 years of age, nonwhite, Catholic, less formally educated, limited to lower family incomes, sexually active, multiparous and married or living in a stable relationship with a partner. This profile is easily explained given that these women lived in the northeastern region of the country, a region historically associated with high levels of social inequality and poverty. It is not uncommon, therefore, even in the twenty-first century, to find young women with very little formal education and no training who are housewives with children.

Our results showed that 75.9% of the participants claimed to have had a Pap test at least once in their lifetime, a rate lower than that reported for women in the cities of Sгo Paulo (86.0%)^17^ and Sгo Luнs do Maranhгo (82.4%)^18^ and in the municipality of Sгo Josй de Mipibu, in the state of Rio Grande do Norte (85.0%).^19^ A significant proportion of the study participants (24.5%) admitted to never having had the test. Our rate was higher than the rates observed in the three studies mentioned above, where the percentages of women who had never undergone the test were 13.9%, 17.6% and 15.0%, respectively. A similarly low rate of 11.2% was reported in Campinas, state of Sгo Paulo.^16^

However, the proportion of women with adequate test practice was 69.6%, thus representing a coverage rate for the test that was slightly higher than that reported for women in Brazil as a whole (66.0%)^20^ and for women in Sгo Josй de Mipibu (64.4%).^19^ Our rate was also very similar to those found in two studies conducted in Pelotas, Rio Grande do Sul,^4^ ^,^ ^21^ in which rates of 68.8% and 68.9%, respectively, were reported. However, our rate was below the coverage rates reported for women in Sгo Paulo (77.3%)^17^ and Sгo Leopoldo, Rio Grande do Sul (85.5%).^22^ The adequacy of test practice demonstrated by our study participants was not influenced by the level of education. This is different from the results obtained in other studies conducted in this country,^16^ ^,^ ^19^ ^,^ ^21^ in which an association was found between the adequacy of this factor and the level of education.

Regarding knowledge, 36.7% of participants demonstrated adequate knowledge of the test, but our rate was lower than that of women in Sгo Josй de Mipibu (46.1%).^19^ We observed that adequate knowledge about the Pap test was found among higher proportions of women who were over the age of 20, ethnically white, married or living in a stable relationship with their partner, more highly educated and sexually active; and who possessed higher family income, and reported medical visits in the year preceding the survey and had children.

For women with a higher level of formal education and greater purchasing power, these results could be explained, at least in part, by the greater access to information about the health benefits of the test that these women had and, possibly, the greater opportunity for them to receive the test. However, the higher rates of adequate knowledge about the test observed among women who were sexually active, who visited a doctor more often and who had children may have been because they sought medical advice more often, either to obtain information regarding contraceptive methods or to receive prenatal care.

With regard to attitudes towards the test, 67.1% of the women interviewed had appropriate attitudes, such that they were able to mention the advantages and benefits of the procedure for their health and to correctly state the reason for periodically undergoing the test. This rate was similar to those reported for women in Sгo Josй de Mipibu (63.3%)^19^ and South Africa (60.6%),^23^ but it was lower than the rate reported for Argentinean women (80.5%)^24^ and higher than the rate observed for women in the Brazilian state of Rio Grande do Sul (45.6%).^25^

Among the women who had an appropriate attitude towards the test, 46.7% considered it necessary to undergo the test periodically to prevent cervical cancer specifically, while 20.5% considered it necessary for preventing cancer, without specifying the type of cancer. A significant proportion of the women studied (9.9%) considered the test necessary but did not know what the benefits of the test were for women's health. This finding suggests that when they learned about the test, the women participating in this study were not well informed regarding the importance of the procedure as a screening method for early diagnosis and treatment of lesions before those lesions progressed to a more malignant form of cancer. Better knowledge of this testing resource is essential for preventing cervical cancer.

The proportion of the women with an appropriate attitude towards the test was significantly higher among those who were already sexually active and married or living in a stable relationship, and among those who consulted a gynecologist during the year prior to the survey. The rates of appropriate attitudes increased proportionally with age, level of education and number of children. These findings were probably due to the greater degree of awareness that these women had about the advantages and benefits of periodic testing, in addition to the more readily available access to information and healthcare services that these women had. Other studies have also reported an association between appropriate attitudes and formal education.^13^ ^,^ ^25^ A study conducted in Campinas,^16^ for example, found an association between appropriate attitudes towards the test and formal education and family income.

In comparing our results regarding test practices with the results available in the literature, we saw that the rate of adequate test practice shown by the women in our study was similar to those described for women in Santo Angelo, Rio Grande do Sul^25^ and Sгo Josй de Mipibu,^19^ but higher than the rates reported for women in South Africa^23^ and Argentina.^24^ The primary reasons given by women for not undergoing the test included absence of symptoms and being too embarrassed to undergo the test. These barriers were similar to those reported in other studies conducted in Brazil^16^ ^,^ ^19^ ^,^ ^25^ and Argentina.^24^

## CONCLUSION

Clearly, health professionals including physicians and nurses play an important role in shaping the knowledge, attitudes and practices of women with regard to the Pap test. Success in this is reflected in the level of adherence to periodic testing among women and, consequently, the coverage rate for the test. Nevertheless, it appears that the forms of communication and/or methodology used by these professionals to inform women about the benefits and advantages of periodic testing may not be sufficiently clear or adequate. However, we cannot rule out the possibility that the time devoted to each consultation and the number of consultations received by these women may not have been sufficient to clarify all matters of concern, or that the women participating in this study had failed to make adequate use of the healthcare services provided. These potential problems require further attention from the municipality's healthcare managers, who need to commit to providing correct information about the test and its advantages and benefits for women's health, in order to improve adherence among the female population, thus meeting the coverage recommendations of the Brazilian Ministry of Health.
